# A Brief Intervention to Increase Uptake and Adherence of an Internet-Based Program for Depression and Anxiety (Enhancing Engagement With Psychosocial Interventions): Randomized Controlled Trial

**DOI:** 10.2196/23029

**Published:** 2021-07-27

**Authors:** Philip J Batterham, Alison L Calear, Matthew Sunderland, Frances Kay-Lambkin, Louise M Farrer, Helen Christensen, Amelia Gulliver

**Affiliations:** 1 Centre for Mental Health Research Research School of Population Health The Australian National University Canberra Australia; 2 The Matilda Centre for Research in Mental Health and Substance Use University of Sydney Sydney Australia; 3 Priority Research Centre for Brain and Mental Health University of Newcastle. Newcastle Australia; 4 Black Dog Institute University of New South Wales Sydney Australia

**Keywords:** implementation, mental health, adherence, uptake, engagement-facilitation intervention, internet, randomized controlled trial

## Abstract

**Background:**

Psychosocial, self-guided, internet-based programs are effective in treating depression and anxiety. However, the community uptake of these programs is poor. Recent approaches to increasing engagement (defined as both uptake and adherence) in internet-based programs include brief engagement facilitation interventions (EFIs). However, these programs require evaluation to assess their efficacy.

**Objective:**

The aims of this hybrid implementation effectiveness trial are to examine the effects of a brief internet-based EFI presented before an internet-based cognitive behavioral therapy self-help program (*myCompass 2*) in improving engagement (uptake and adherence) with that program (primary aim), assess the relative efficacy of the *myCompass 2* program, and determine whether greater engagement was associated with improved efficacy (greater reduction in depression or anxiety symptoms) relative to the control (secondary aim).

**Methods:**

A 3-arm randomized controlled trial (N=849; recruited via social media) assessed the independent efficacy of the EFI and *myCompass 2*. The *myCompass 2* program was delivered with or without the EFI; both conditions were compared with an attention control condition. The EFI comprised brief (5 minutes), tailored audio-visual content on a series of click-through linear webpages.

**Results:**

Uptake was high in all groups; 82.8% (703/849) of participants clicked through the intervention following the pretest survey. However, the difference in uptake between the EFI + *myCompass 2* condition (234/280, 83.6%) and the *myCompass 2* alone condition (222/285, 77.9%) was not significant (n=565; *χ*^2^_1_=29.2; *P=*.09). In addition, there was no significant difference in the proportion of participants who started any number of modules (1-14 modules) versus those who started none between the EFI + *myCompass 2* (214/565, 37.9%) and the *myCompass 2* alone (210/565, 37.2%) conditions (n=565; *χ*^2^_1_<0.1; *P=*.87). Finally, there was no significant difference between the EFI + *myCompass 2* and the *myCompass 2* alone conditions in the number of modules started (*U*=39366.50; *z*=−0.32; *P=*.75) or completed (*U*=39494.0; *z*=−0.29; *P=*.77). The *myCompass 2* program was not found to be efficacious over time for symptoms of depression (*F*_4,349.97_=1.16; *P=*.33) or anxiety (*F*_4,445.99_=0.12; *P=*.98). However, planned contrasts suggested that *myCompass 2* may have been effective for participants with elevated generalized anxiety disorder symptoms (*F*_4,332.80_=3.50; *P=*.01).

**Conclusions:**

This brief internet-based EFI did not increase the uptake of or adherence to an existing internet-based program for depression and anxiety. Individuals’ motivation to initiate and complete internet-based self-guided interventions is complex and remains a significant challenge for self-guided interventions.

**Trial Registration:**

Australian New Zealand Clinical Trials Registry ACTRN12618001565235; https://www.anzctr.org.au/Trial/Registration/TrialReview.aspx?id=375839

## Introduction

### Background

Implementation science focuses primarily on implementing novel evidence-based interventions in existing health care systems [1]. However, this approach does not involve people who do not engage in health services. The adoption of evidence-based interventions outside of health care settings is particularly pertinent to mental health. The burden of mental ill health and prevalence of mental health problems are high worldwide [2-5]. However, up to two-thirds of people with mental health conditions do not seek formal evidence-based treatment [6], with many citing stigma [7] and cost [8] as barriers to seeking face-to-face treatment. One proposed solution for addressing this unmet need is to offer evidence-based, low-intensity, self-guided e–mental health (E–MH) programs broadly to people in the community with mild-to-moderate symptoms [9], which could improve the efficiency of services and increase the availability of specialist care for those with more severe mental health problems [10]. However, limited studies have investigated strategies to reduce the barriers to the implementation of self-guided psychosocial programs within the wider community.

Considerable evidence has demonstrated that self-guided internet-based programs are effective in preventing and treating symptoms of common mental health problems such as depression [10,11]. Despite significant benefits to the individual and the ability to directly address perceived key barriers to accessing treatment [12], community uptake of these programs remains poor [12,13], which strongly suggests that there must be other barriers to using internet-based treatment. Research on a self-guided E–MH program for depression found that half of its unique visitors from the general community (N=194,840) did not register for the program [14], and only half of those who registered subsequently engaged in any of the program’s modules. These findings indicate that those who commenced engaging in programs likely experienced barriers to adherence. Primary care research has reported even lower rates of uptake for E–MH programs of between 3% and 25% [15]. Successful targeting and reduction of barriers to uptake by even a small amount may substantially increase the number of people receiving evidence-based treatment.

### Barriers to Engaging and Adhering to E–MH Treatment

There are many reported barriers to the uptake of E–MH programs [16]. These include a preference for face-to-face therapy over E–MH programs [12,17,18]; the common perception that internet-based therapies are not as effective as face-to-face therapy [17]; and concerns about issues such as data security, limited familiarity with E–MH programs, negative attitudes toward seeking help in general, or anxiety around using the internet [9,10,15-17,19]. Similarly, adherence to E–MH programs is also a significant challenge, particularly in the community outside of research settings [14,20]. There are also many proposed reasons for low levels of adherence. Some are positive, such as the individual receiving a sufficient dosage for symptom remission. Others are neutral, such as a lack of treatment needs (eg, healthy users). Negative reasons for low adherence include low motivation, a lack of perceived improvement, or the failure of the program to adequately engage the user or meet their needs or expectations [14,20-22].

Many of these attitudinal barriers are modifiable and might be offset by benefits such as increased privacy [23], high fidelity of delivery, and increased accessibility [24]. It is proposed that challenging some of these potentially modifiable barriers before an individual began an E–MH program is an implementation strategy that might increase subsequent engagement with the program [15]. Interventions based on this concept are called acceptance-facilitation interventions (AFIs). These interventions are supported by behavior change theories [25,26], which suggest that improving attitudes and social norms for the use of E–MH interventions will lead to greater acceptability and uptake [27]. AFIs comprise a brief package of information designed to target some of the noted potential barriers to program acceptability, with the ultimate goal of increasing both uptake and adherence (engagement) to these programs [15]. Two previous randomized controlled trials (RCTs) of AFIs showed improved acceptability attitudes of E–MH programs for people with chronic pain [28], diabetes [29], and depression [15]. However, all three trials measured attitudes immediately following the AFI. Conversely, a study that followed was not able to replicate this improved acceptability following the presentation of an E–MH program for chronic pain [30]. This study was unable to improve the uptake rate or adherence to the subsequent E–MH program. The authors concluded that perhaps the AFI did not influence intervention uptake and adherence because it only targeted acceptability [30]. There is a need to develop and evaluate brief interventions that target other factors related to uptake and adherence, in addition to attitudes regarding acceptability.

### Aims

Further research is clearly needed to evaluate AFIs, or more broadly, interventions that target engagement with internet-based psychosocial programs, which we label engagement facilitation interventions (EFIs). This study adopts a model of engagement [31,32] that includes both the initiation of the program (uptake) and its ongoing use (adherence). Thus, EFIs target both uptake and adherence to programs. Currently, no known studies have examined the utility of an EFI in increasing the uptake of and adherence to an existing, publicly available E–MH program. This study describes the results of a 3-armed hybrid implementation effectiveness RCT [33] evaluating the efficacy of a newly developed EFI on uptake and engagement with an existing E–MH program. The primary aim of the study is to test the effects of the EFI on uptake and engagement; the secondary aim is to test the relative efficacy of the internet-based cognitive behavioral therapy intervention with and without the EFI and test whether increased uptake and adherence were associated with greater efficacy; and the exploratory aim is to identify moderators of differential uptake, adherence, and efficacy, contingent on between-group differences in these outcomes. The EFI used in this study was developed through an iterative participatory design process with people who have lived experience of depression or anxiety to ensure that it met their needs, while accounting for key barriers to E–MH implementation identified in previous empirical studies. Given that the EFI content was designed to target both acceptability and adherence to the E–MH program, our primary hypothesis was that the EFI would increase participants’ uptake of the E–MH program, defined as the initiation of at least one module of the E–MH program, and increase adherence, defined as a greater number of modules completed.

## Methods

### Trial Design

A 3-arm RCT, called the *Enhancing Engagement with Psychosocial Interventions* (EEPI) trial, assessed the independent efficacy of the EFI and *myCompass 2* by comparing the conditions of (1) *myCompass 2* + EFI, (2) *myCompass 2* (alone), and (3) an attention control condition.

### Ethics Approval

The ethical aspects of this research were approved by The Australian National University Human Research Ethics Committee (protocol number 2018/257).

Multimedia Appendices 1 and 2 present the CONSORT (Consolidated Standards of Reporting Trials) checklists for reporting randomized trials [34].

### Interventions

#### The EFI

**Figure 1:**
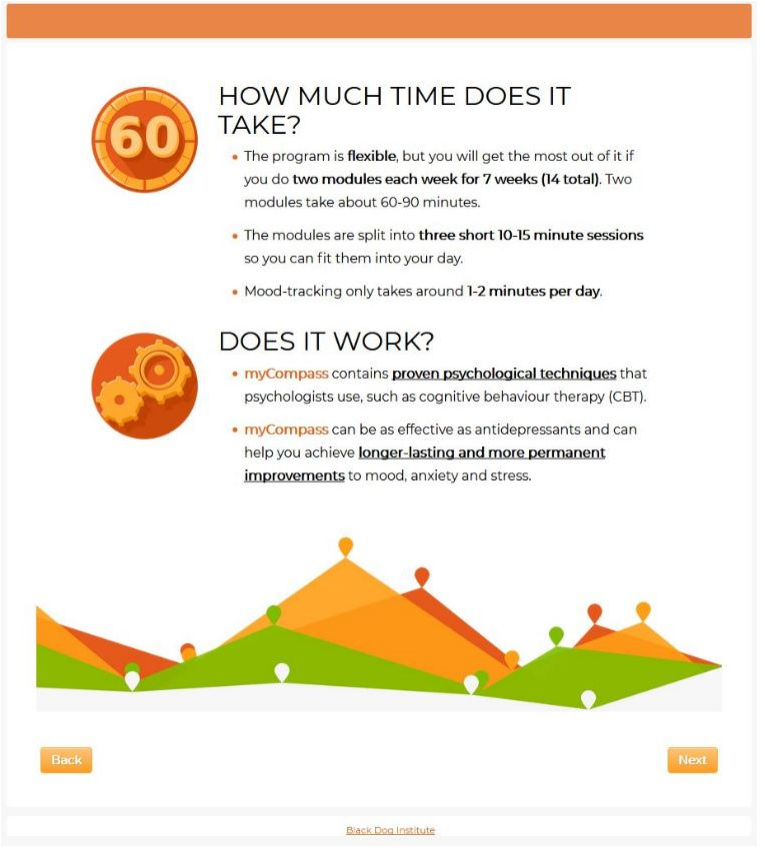
Engagement facilitation intervention content example page.

The EFI comprised brief, tailored, written, and audio-visual content (approximately 5 minutes) presented to participants in the EFI condition (EFI *+ myCompass 2*) on a series of click-through linear webpages. The participants in this condition viewed the EFI after they were randomized and before commencing the E–MH program for depression and anxiety (*myCompass 2*). The EFI was delivered on an internet-based platform that also housed the surveys and control group content. Figure 1 presents some examples of the EFI. The look and feel of the EFI was based on the design of the *myCompass 2* program to create a seamless flow of the EFI intervention into *myCompass 2*.

We used principles of participatory design to create the EFI, as these can improve the perceived relevance and uptake of interventions for end users [35]. We developed the EFI through a focus group study [36] of community members who had personal lived experience of depression or anxiety, or both (n=24, four groups; male=3, female=21; see the study by Gulliver et al [36] for further details on the EFI and its development). As noted in our study [36], very few males participated in these groups. Community members in the groups suggested that the EFI content should target barriers to using these programs through the provision of personalized symptom level feedback to demonstrate program needs, information about the program content, data security, program efficacy, and finally content challenging potential social norms around using E–MH programs (eg, the belief that others do not use E–MH programs). In particular, we used written and video content that was informed by theory that examines how social norms influence the acceptability of E–MH programs (eg, “online programs and apps are being increasingly used by people in the community to look after their mental health in their own time”) [15,25,27].

The EFI comprised the following components:

1. Feedback about the participant’s symptom levels (visual graph) and a written description of the benefits of participating in E–MH programs, tailored to symptom levels.
2. Written information about the efficacy of the E–MH program, its content, the time commitment involved, and its data security.
3. Two testimonials (presented in a single 1-minute video) outlining the benefits of E–MH programs to provide information and normalize participation in internet-based, self-guided therapy interventions.

#### myCompass 2

The E–MH program used was an updated version of the *myCompass* program that we named for this study as *myCompass 2. myCompass* has previously been demonstrated to reduce symptoms of depression and anxiety in two community samples (n*=*89; n=720) [37,38]. The *myCompass 2* program is a fully automated and interactive self-help program that is free to the end user and was designed for people with mild-to-moderate symptoms of stress, anxiety, and depression. It is delivered without therapist assistance and can be accessed privately, at any time, on a variety of platforms, including mobile phones, tablets, and computers.

The *myCompass 2* program is similar to its predecessor and contains 14 modules derived primarily from cognitive behavioral therapy, problem-solving therapy, interpersonal psychotherapy, and positive psychology. The program was delivered over 7 weeks to enable sufficient time to complete the 14 modules. The user is expected to complete two modules per week. However, symptom reduction may occur with the completion of fewer modules [38,39]. Each module takes 30-45 minutes to complete; thus, the expected commitment of the program is approximately 60-90 minutes per week. Half (7/14, 50%) of the modules provided core transdiagnostic cognitive behavioral therapy, whereas the other half provided content targeting specific concerns related to mental health (eg, sleep). Additional interactive features are also included such as quizzes providing real-time self-monitoring of thoughts, feelings, and behaviors; self-monitoring reminders, feedback, facts, and mental health care tips or motivational statements provided via SMS text messaging or email. Similar to the original *myCompass* [38], *myCompass 2* was designed to be tailored to the user’s needs. The program screens the user and then directs them to modules that are likely to be suitable for them. The clinical approaches of the original *myCompass* and *myCompass 2* programs were identical. However, the manner in which the content is delivered, the design of the program, and the user experience were all upgraded for *myCompass 2,* focusing on personalization and ease of use. This process was influenced by input from clinicians, consumers, and information technology specialists, resulting in the following key differences: (1) faster and easier sign up for users, (2) a more comprehensive user dashboard with more options for personalization, (3) modules that are more clearly displayed and easier to navigate, and finally (4) more comprehensive symptom trackers that are easier to personalize for the user.

#### Attention Control Condition (HealthWatch)

The attention control condition (*HealthWatch*) has previously been shown to have high credibility [40,41]. It was approximately matched to the *myCompass 2* program for the time taken to complete and comprised 14 brief modules of written information offered over 7 weeks. The information was taken from public domain health and lifestyle information and was deliberately unrelated to mental health. Module topics included *Keeping bones strong and healthy*, *Your microbes and you*, and *The power of your pancreas*. At the end of the 6-month trial period, after the follow-up data were collected, the control group was sent information about how to access the *myCompass 2* program if they wished.

### Procedure

#### Recruitment

Participants were recruited from the general community via a social network, Facebook, from January to March 2019. As outlined in the study protocol [42], the recruitment target of at least 231 participants per condition (n=693) to meet the power requirements for our primary hypotheses (see *Outcomes and Data Analysis* section for further details), was met within 2 months. All follow-up data were collected in November 2019. Facebook was used to ensure that a broad cross-section of the community could be reached and to maintain ecological validity, as internet-based interventions are often marketed on the web directly to consumers. We set up a Facebook page to describe the study and used paid Facebook advertisements using nature imagery (eg, trees, waterfalls) that asked, “Want to learn more about your mental health? Complete a survey and 7-week online program now” along with the ethics approval information listed earlier. We also ran a concurrent advertisement targeting male Facebook users only and using typical masculine-targeted imagery (eg, images of road journeys). This was to increase male participants and address the commonly higher ratio of female to male participants in internet-based mental health trial research [43]. To increase the representation of males in the study, we continued this advertisement targeting male participants after meeting the original target sample of 693 participants after the first month of recruitment. This advertising strategy was slower and took another month; however, at that time, we increased the percentage of males in the study from 12% to 21.9%. Participants who then clicked *sign up* were directed to the information and consent page, where they were provided the key details of the study and asked to provide their consent to participate on the web.

Participants were invited to read the information sheet and consent to participate before completing the screening measures. After consenting, participants were screened using a two-stage screening process. First, they read a list of eligibility criteria that they had to endorse to be eligible for the trial. These were as follows: (1) had not previously used the *myCompass* web-based program, (2) were not currently receiving psychological therapy, (3) had not made a suicide plan in the past month, (4) had not been diagnosed with psychosis or bipolar disorder, (5) were aged 18 years or older, and (6) were currently living in Australia. The second part of the screening process involved completing the Generalized Anxiety Disorder 7-item (GAD-7) [44] and Patient Health Questionnaire-9 (PHQ-9) [45] instruments. Consistent with the approach that internet-based programs are highly suitable for those with mild-to-moderate symptoms in the community [10], potential participants were eligible if they reported current symptoms of depression, anxiety, or both in the mild-to-moderate range (score 5-14) using the screening instruments (PHQ-9 and GAD-7) described as follows. If they scored too low (0-4) on both instruments or too high (15+) on either instrument, they were not eligible. Those who were screened as not eligible at any point in the two-stage screening process were excluded from the study and provided with relevant mental health resources. All participants endorsing the suicide screening item of the PHQ-9 were provided with a prompt that asked them to telephone Lifeline, the Suicide Call Back Service, or 000 in the case of emergency.

#### Treatment Allocation

We delivered the trial using the digital infrastructure portal of the Black Dog Institute, Sydney, Australia. The portal allowed for computer-generated random allocation, automatic assessments, intervention materials, and reminders to be delivered seamlessly to the participants. The portal collected web use data automatically for all conditions, allowing us to assess participants’ uptake and usage (adherence) of their assigned program. After completing the two-stage screening process, participants provided an email address and selected a password they could use to log in to access their assigned treatment. Participants were then randomized to one of the three conditions, using computer-based randomization stratified by general psychological distress symptom severity (as measured by the Distress Questionnaire-5 [DQ5] [46], at pretest score 5-13 vs 14-25), age (18-45 vs ≥46 years), and gender (female or male; permuted block randomization, block size of 6 within each stratum) to ensure balance across conditions. *Prefer not to answer* and *other* were categorized as the group expected to be smaller (ie, male gender and age ≥46 years). This was completed using a computerized randomization algorithm embedded in the trial portal. Those who were assigned to the *myCompass 2* conditions (conditions 1 and 2) were required to log in again when automatically redirected to the *myCompass 2* program.

#### Data Collection

The intervention period ran for 7 weeks. By using their email and password, participants were able to access their assigned programs as much or as little as they preferred. Through the automated system, weekly reminder emails were sent to participants to encourage them to engage with the *myCompass 2* (conditions 1 and 2) or the attention control websites (condition 3). Participants were also sent emails to complete the 7-week posttest and 6-month follow-up surveys. Participants were sent reminder emails to complete if they had not completed the questionnaire after 1 and 2 weeks. Individual participant access to surveys was closed 4 weeks after the initial email was sent to each participant.

#### Incentives

We emailed participants with small incentives in the form of e-gift cards for the completion of each internet-based assessment across all conditions (posttest incentive: Aus $15 [US $11.3]; follow-up incentive: Aus $25 [US $18.8]), regardless of their level of engagement with the intervention. These incentives were considered a token of appreciation for the participants’ time and effort for each survey.

#### Blinding

The trial was double-blinded. The participants were blinded to whether they received active or attention control interventions. They were informed that they would be randomized to receive one of three programs: (1) strategies for challenging unhelpful thoughts and behaviors (*myCompass 2*), (2) education about internet-based interventions plus program (EFI + *myCompass 2*), or (3) general health and lifestyle information (attention control). They were not provided with information about which of these interventions was expected to be the most effective. Assessments were also blinded, as they were self-report. The statistician performing the analyses was also blinded to the conditions.

### Outcomes and Data Analysis

#### Primary Hypotheses (Aim 1)

We hypothesized that uptake (initiation of at least one module) would be higher in the EFI + *myCompass 2* condition than in the *myCompass 2* alone condition (H1). We also expected greater adherence to be observed (ie, higher number of modules completed) in the EFI + *myCompass 2* condition relative to *the myCompass 2* alone condition (H2).

#### Secondary Hypotheses (Aim 2)

We expected that efficacy (reduction in symptoms of depression and anxiety) would be higher in the two active *myCompass 2* intervention conditions than the *HealthWatch* attention control condition at posttest and 6-month follow-up (H3). Finally, we also expected that efficacy would be higher in the EFI + *myCompass 2* condition than in the *myCompass 2* alone condition at posttest and follow-up, and this difference would be mediated by adherence to the program (H4).

#### Exploratory Hypotheses

We expected that uptake (H1), adherence (H2), and efficacy (H3) would be moderated by a range of sociodemographic and psychological characteristics, including gender, age, cultural and linguistic background, education, social support, symptoms of depression and anxiety, acceptability of psychosocial internet-based programs, attitudes toward professional psychological treatment, familiarity and use of technology, personality, stigma, and mental health literacy (H5). We also predicted that secondary indices of efficacy (reductions in suicidality, distress, and disability; increases in acceptability of internet-based psychosocial interventions and quality of life) would be greatest in the EFI + *myCompass 2*, followed by *myCompass 2* alone, which would also outperform the attention control condition (H6).

### Power

To detect a significant difference in uptake (H1) from 50% to 65% (a conservative baseline based on previous research [47]), with conservative difference based on previous work by Ebert et al [15] with 90% power required a sample of 231 per condition (α=.05). To detect a difference in adherence (H2), assuming a small effect of *f=*0.19 (the estimated median effect from previous research [47]) between active conditions required a sample of 111 per condition. For the efficacy hypotheses (H3 and H4), a sample of 110 per condition was required to find an effect size of *f*=0.18 (based on Proudfoot et al [38]) between active conditions relative to control over the three assessment time points (baseline, post, and 6-month follow-up) with 90% power (α=.05; *r*=0.5; between repeated measures). We allowed up to 30% attrition from the posttest assessments. Thus, a minimum sample size of 158 per condition was required. We aimed to recruit a sample of n=693, which was based on the largest estimate of N required (n=231 per condition). This study achieved an adequate sample size (N=849) to meet the power requirements.

### Measures

#### Demographic Characteristics

The following demographic characteristics were assessed: gender, age category, language spoken at home, level of education (primary school, some secondary school or year 10 equivalent, year 12, certificate level I-IV, diploma or associate degree, bachelor degree, graduate diploma or graduate certificate, master’s degree, or doctoral degree), employment status (full-time, part-time or casual, unemployed, not working due to study or maternity leave, retirement, etc), and region or area of residence (metropolitan area, regional area, or rural or remote area). Other assessments including previous psychological treatment, mental health literacy, and stigma were also measured as per the protocol [42] but were not included here because we were not able to investigate H5 due to null findings on H1, H2, and H3.

#### Depression Symptoms

The PHQ-9 [45] was used to assess the symptoms of depression. This scale consists of nine items that assess the frequency of Diagnostic and Statistical Manual of Mental Disorders (DSM)-IV symptoms of major depression during the past two weeks. Participants rated items on a 4-point scale ranging from 0 (not at all) to 3 (nearly every day). Scores from each item were summed to produce an overall severity score, which ranges from 0 to 27. Higher scores indicate a higher severity of depression symptoms. The PHQ-9 has previously shown good sensitivity and specificity for detecting major depression in clinical and general population samples and has also demonstrated sensitivity to change over time [48]. Internal reliability using Cronbach α was .45 (N=849) at pretest. However, Cronbach α is not recommended when participants’ scores fall within a restricted range [49], such as in this study where we recruited participants with symptom scores of 5-14 only for both PHQ-9 and GAD-7. In the posttest, when the range was broader (0-26), Cronbach α for the PHQ-9 was .85.

#### Anxiety Symptoms

We used the GAD-7 to assess anxiety symptoms. The GAD-7 scale comprises seven items that correspond to the DSM-IV and DSM-V criteria for generalized anxiety disorder [44]. Participants rated items on the same 4-point scale as the PHQ-9. Scores for each item were summed and ranges from 0 to 21, with higher scores indicating greater symptom severity. Previous research has demonstrated that the GAD-7 has good psychometric properties in general population samples, has similar properties in detecting changes to the PHQ-9, and provides accuracy compared with clinical diagnosis [48,50]. This study sample Cronbach α was .75 (N=849) at pretest, but as above, this may not be a reliable estimate. In the posttest, Cronbach α was .88 at posttest.

#### General Psychological Distress

We used the DQ5 [46] to measure general psychological distress. Given that DQ5 provides coverage of both depression and anxiety symptoms, for the purpose of this study, this measure was also used to stratify participants at randomization. We selected the case finding cut-off point of ≥14 (lower distress=5-13; higher distress=14-25) for stratification [46]. The DQ5 comprises five items that ask respondents to indicate the frequency of a range of distressing situations, thoughts, and feelings over the previous 30 days using a 5-point Likert-type scale (1=never, 2=rarely, 3=sometimes, 4=often, and 5=always). Scores are summed, and total scores range from 5 to 25, with higher scores indicating more severe levels of general psychological distress. Previous studies have demonstrated that DQ5 displays high internal consistency and external validity [46,51]. The current sample DQ5 Cronbach α was .72 at pretest in the restricted sample of individuals randomized into the study.

#### Acceptability of Internet-Based Psychosocial Programs

The acceptability of internet-based programs was assessed using items developed and compiled by Ebert et al [15], based on the Unified Theory of Acceptance and Use of Technology. This measure assesses acceptability, with each item rated on a 5-point scale ranging from *totally disagree* to *totally agree*. For example, one item is as follows:

If I was suffering from psychological strain such as enduring lowered mood, loss of interest and lowered energy, sleeping problems, rumination, loss of joy in life...I could imagine trying out an internet-based intervention for mental health problems.

Scores are summed (range 4-20); higher scores indicate higher acceptability of internet-based programs. This scale has acceptable internal consistency [15]. Out of the eight total scales created by Ebert et al [15], we selected the following three scales for this study: *performance expectancy* (4 items from Wilson and Lankton [52] and Schomerus et al [53], example item: “Using an internet-based training would reduce my mental health problems”), *effort expectancy* (3 items, from the studies by Wilson et al [52] and Schomerus et al [53], example item: “Using an internet-based depression intervention would be an easy task for me”), and finally *concerns regarding data security* (2 items, developed by Ebert et al [15], example item: “When participating in an online-training I would trust, that all information I disclose would be treated in strict confidence”). All items were rated on the same 5-point scale as previously described.

#### Suicidal Ideation

We used five suicide-specific items from the Psychiatric Symptom Frequency scale [54] to measure suicidal ideation. Items measure suicidal ideation and suicidal behavior, using items that cover suicidal thoughts, plans, and attempts. Respondents chose yes or no to indicate whether any of these aspects of suicidal ideation or behavior were present in the previous six months; higher scores indicated higher severity of suicidal ideation and actions. Psychiatric Symptom Frequency scale suicide items display high internal reliability and validity [54]. These items were assessed at the pretest and 6-month follow-up only.

#### Disability or Days Out of Role

Disability and the days out of role were measured by two items. The first assessed the number of days out of role: “In the last 30 days, how many days were you totally unable to work, study, or manage your day-to-day activities because of emotional problems (such as feeling depressed or anxious)?” The second assessed days of disability: “Aside from those days, in the last 30 days, how many days were you able to work, study, or manage your day-to-day activities but had to cut back on what you did or did not get as much done as usual because of emotional problems?”

#### Quality of Life

We used the European Health Interview Survey Quality of Life 8-item index (EUROHIS-QOL) [55] to measure quality of life. This scale comprises eight items measuring the psychological, physical, social, and environmental components of quality of life. Two examples include, “How would you rate your quality of life?” and “How satisfied are you with your ability to perform your daily activities?” Respondents rated the items on a 5-point scale, with response categories ranging from very dissatisfied to very satisfied, very poor to very good, and not at all to completely. A total score ranging from 0 to 32 is produced by summing the scores from each item; higher scores indicate a higher perceived quality of life. Previous research has shown adequate internal consistency for EUROHIS-QOL in multiple samples [55,56].

#### Perceived Reasons for Nonadherence

We also asked participants at the end of the posttest survey to complete a measure of self-reported adherence by asking, “Did you complete all of the program?” For those that responded “No,” we asked, “What were some of the reasons you didn’t complete the program?”

### Analyses

#### Primary Outcomes (Aim 1)

The primary outcomes were uptake and adherence to the *myCompass 2* program. Uptake (H1) was assessed as the number of individuals who accessed at least one therapeutic module of the program. We compared the rate of uptake in EFI + *myCompass 2* to that in the *myCompass 2* alone condition using a chi-square test. Adherence (H2) was assessed using a Mann-Whitney *U* test to compare the number of modules that started and completed *myCompass 2* during the intervention period of 7 weeks. We selected modules completed as they captured the dosage of the therapeutic content received. We also examined qualitative data on self-reported reasons for nonadherence using thematic analysis.

#### Secondary Outcomes (Aim 2)

Efficacy (H3 and H4) was assessed on the basis of reduced symptoms of depression (PHQ-9 [45]) and GAD-7 [44] at the posttest and 6-month follow-up. Comparisons of efficacy were made between participants in each of the active intervention conditions and those in the attention control condition. We analyzed the secondary efficacy outcomes using an intention-to-treat framework, using data collected from the three measurement occasions (pretest, posttest, and 6-month follow-up). We calculated the effects with IBM SPSS 26.0 for Windows (IBM Corporation) using mixed model repeated measures (MMRM) analyses of variance, conservatively estimated using unstructured covariance matrices. Mixed models techniques incorporate all available data, including data from participants who did not complete assessments at posttest or follow-up, under the assumption that data were missing at random [57].

#### Exploratory Outcomes

The MMRM processes described above were also used to examine the effects of the interventions on secondary efficacy outcomes (H6). The exploratory logistic regression and negative binomial regression models, and growth mixture model analyses (planned for H5) [42] did not proceed due to null findings on H1, H2, and H3.

## Results

### Participants

**Figure 2:**
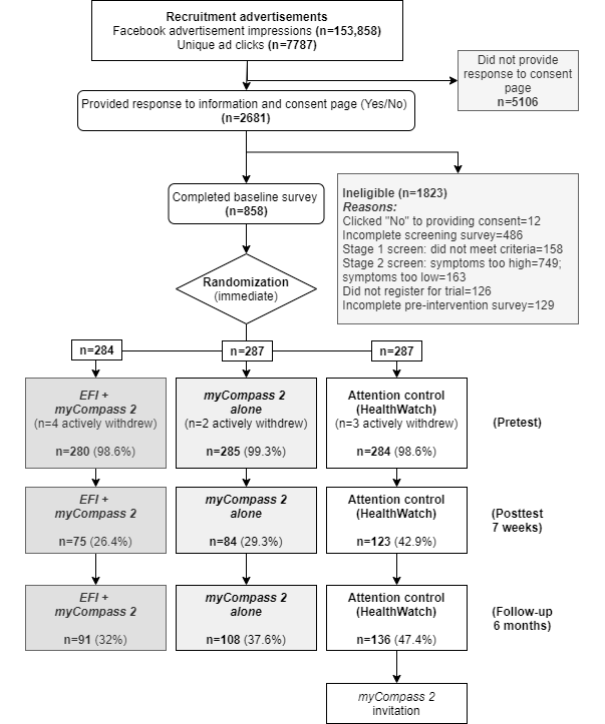
Flow of participants through the trial. Percentages are from the total randomized. EFI: engagement facilitation intervention.

A total of 858 participants met the eligibility criteria, agreed to participate in the trial, and completed the baseline assessment. Nine participants withdrew over the course of the study, and these were evenly spread across the three conditions. Figure 2 shows a CONSORT diagram of participant flow throughout the study. Overall retention rates from randomization were 32.9% at posttest and 39% at follow-up.

Table 1 presents the participants’ characteristics. There were no significant differences between the conditions at pretest for any of the characteristics.

**Table 1:** Characteristics of participants included in this study (N=849).

Characteristic			Intervention arm				Attention control (n=284)		Total (N=849)
			EFI^a^ + *myCompass 2* (n=280)		*myCompass 2* alone (n=285)				
**Age category (years)** **, n (%)**									
	18-25	30 (10.7)		24 (8.4)		25 (8.8)		79 (9.3)	
	26-35	55 (19.6)		50 (17.5)		54 (19)		159 (18.7)	
	36-45	84 (30)		79 (27.7)		84 (29.6)		247 (29.1)	
	46-55	68 (24.3)		86 (30.2)		60 (21.1)		214 (25.2)	
	56-65	35 (12.5)		39 (13.7)		44 (15.5)		118 (13.9)	
	≥66	8 (2.9)		7 (2.5)		17 (6)		32 (3.8)	
**Gender** **, n (%)**									
	Male	57 (20.4)		61 (21.4)		68 (23.9)		186 (21.9)	
	Female	217 (77.5)		217 (76.1)		212 (74.6)		646 (76.1)	
	Other	4 (1.4)		2 (0.7)		2 (0.7)		8 (0.9)	
	Prefer not to answer	2 (0.7)		5 (1.8)		2 (0.7)		9 (1.1)	
**Highest level of education** **, n (%)**									
	High school or less	52 (18.6)		49 (17.2)		54 (19)		155 (18.3)	
	Certificate or diploma	108 (38.6)		93 (32.6)		103 (36.3)		304 (35.8)	
	Bachelor’s degree	67 (23.9)		71 (24.9)		71 (25)		209 (24.6)	
	Postgraduate degree or diploma	51 (18.2)		72 (25.3)		55 (19.4)		178 (21)	
	Prefer not to answer	2 (0.7)		0 (0)		1 (0.4)		3 (0.4)	
**Employment** **, n (%)**									
	Full-time	96 (34.3)		96 (33.7)		100 (35.2)		292 (34.4)	
	Part-time or casual	99 (35.4)		83 (29.1)		91 (32)		273 (32.2)	
	Unemployed	35 (12.5)		44 (15.4)		29 (10.2)		108 (12.7)	
	Not working (eg, study or maternity leave)	46 (16.4)		56 (19.6)		59 (20.8)		161 (19)	
	Prefer not to answer	4 (1.4)		6 (2.1)		5 (1.8)		15 (1.8)	
**Language** **, n (%)**									
	English	272 (97.1)		275 (96.5)		273 (96.1)		820 (96.6)	
	English and other or other language only	8 (2.9)		10 (3.5)		11 (3.9)		29 (3.4)	
**Location** **, n (%)**									
	Metropolitan	115 (41.1)		123 (43.2)		122 (43)		360 (42.4)	
	Regional	132 (47.1)		119 (41.8)		121 (42.6)		372 (43.8)	
	Rural or remote	32 (11.4)		42 (14.7)		41 (14.4)		115 (13.5)	
	Prefer not to answer	1 (0.4)		1 (0.4)		0 (0)		2 (0.2)	
**Symptom measures, mean (SD)**									
	Anxiety (GAD-7^b^)	7.63 (3.42)		7.28 (3.27)		7.71 (3.37)		7.54 (3.36)	
	Depression (PHQ-9^c^)	9.82 (2.77)		9.64 (2.70)		9.49 (2.92)		9.65 (2.80)	
	General psychological distress (DQ5^d^)	14.50 (2.96)		14.39 (3.07)		14.42 (2.87)		14.44 (2.97)	
	Acceptability (UTAUT^e^)	14.49 (3.18)		14.42 (3.01)		14.91 (2.87)		14.61 (3.03)	
	Performance expectancy (UTAUT)	13.53 (2.67)		13.41 (2.67)		13.89 (2.70)		13.61 (2.69)	
	Effort expectancy (UTAUT)	10.66 (2.19)		10.67 (2.12)		10.54 (1.98)		10.62 (2.09)	
	Data security concerns (UTAUT)	7.48 (1.86)		7.30 (1.96)		7.37 (1.85)		7.38 (1.89)	
	Days out of role	3.04 (4.49)		4.14 (5.92)		3.62 (5.65)		3.61 (5.41)	
	Days cut down	9.01 (8.33)		9.51 (8.65)		7.60 (7.32)		8.71 (8.15)	
	Quality of life (EUROHIS-QOL^f^)	24.19 (5.15)		23.57 (5.13)		24.46 (5.68)		24.07 (5.33)	
	Suicidal ideation (PSF^g^)	0.96 (1.21)		0.95 (1.21)		0.84 (1.09)		0.92 (1.17)	

^a^EFI: engagement facilitation intervention.

^b^GAD-7: Generalized Anxiety Disorder-7 item.

^c^PHQ-9: Patient Health Questionnaire-9.

^d^DQ5: Distress Questionnaire-5.

^e^UTAUT: Unified Theory of Acceptance and Use of Technology.

^f^EUROHIS-QOL: European Health Interview Survey Quality of Life 8-item index.

^g^PSF: Psychiatric Symptom Frequency scale.

### Missingness

A chi-square test indicated a significant difference between conditions in completion of posttest assessments (N=849; χ^2^_2_=19.3; *P*<.001), and 6-month follow-up assessments (N=849; χ^2^_2_=13.2; *P=*.001). Examination of the standardized residuals indicated that there were fewer participants missing from the control group, and a greater number of participants were missing from the EFI + *myCompass 2* condition at both the posttest and follow-up.

### Uptake (H1)

Table 2 shows that overall uptake of the programs was very high (703/849, 82.8%). A chi-square analysis using Fisher's exact test demonstrated that the difference in uptake between conditions overall was significant (N=849; *χ*^2^_2_=8.3; *P=*.02). However, the difference in uptake between the EFI + *myCompass 2* (472/565, 83.6%) and the *myCompass 2* alone (440/565, 77.9%) conditions was not significant (n=565; *χ*^2^_1_=29.2; *P=*.09). There was also no significant difference in the proportion of participants who did and did not start a module in the *myCompass 2* program between the EFI + *myCompass 2* (214/565, 37.9%) and the *myCompass 2* alone (210/565, 37.2%) conditions (n=565; *χ*^2^_1_<0.1; *P=*.87).

**Table 2:** Uptake and adherence data (N=849).

Outcome			Intervention arm, n (%)				Total (N=849), n (%)
			EFI^a^ + *myCompass 2* (n=280)	*myCompass 2* alone (n*=*285)	Attention control (n=284)		
**Uptake (clicked through to program following pretest survey)^b^**							
	Yes		234 (83.6)	222 (77.9)	247 (87)	703 (82.8)	
	No		46 (16.4)	63 (22.1)	37 (13)	146 (17.2)	
**Uptake (number of modules started)**							
	0		174 (62.1)	179 (62.8)	36 (12.7)	389 (45.8)	
	**1-14**		106 (37.9)	106 (37.2)	248 (87.3)	460 (54.2)	
		1-2	69 (24.6)	74 (26)	232 (81.7)	375 (44.2)	
		3-6	31 (11.1)	27 (9.5)	8 (2.8)	66 (7.8)	
		7-14	6 (2.1)	5 (1.8)	8 (2.8)	19 (2.2)	
**Adherence (number of modules completed)^c^**							
	0		216 (77.1)	223 (78.2)	36 (12.7)	475 (55.9)	
	**1-14**		64 (22.9)	62 (21.8)	248 (87.3)	374 (44.1)	
		1-2	38 (13.6)	36 (12.6)	232 (81.7)	306 (36)	
		3-6	20 (7.1)	22 (7.7)	8 (2.8)	50 (5.9)	
		7-14	6 (2.1)	4 (1.4)	8 (2.8)	18 (2.1)	

^a^EFI: engagement facilitation intervention.

^b^One participant in the control group subsequently clicked through to the intervention 1 month after the survey was completed; thus, they are not included in the figures for uptake but are included in the modules started or completed and all their related statistical tests.

^c^Control group participants were able to access their program directly following the pretest survey without logging in, and they viewed a single page to complete a module; thus, the modules started or completed for this group are identical and not directly comparable with the intervention conditions.

### Adherence (H2)

**Figure 3:**
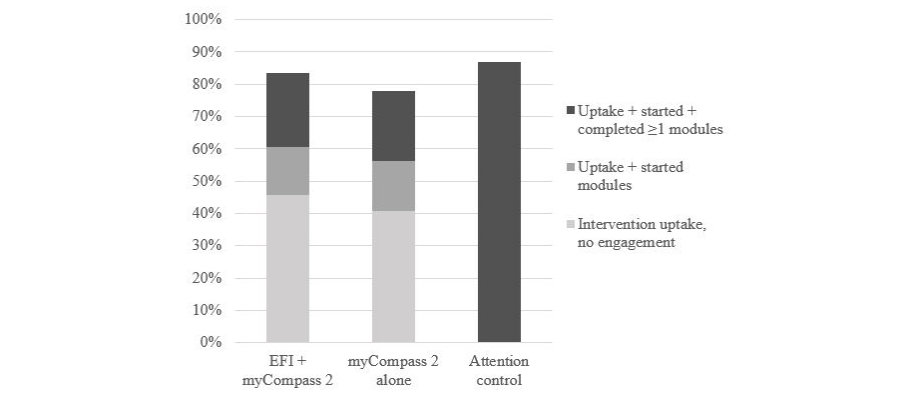
Cumulative uptake and adherence to the programs by condition. EFI: engagement facilitation intervention.

The overall adherence to the *myCompass 2* program was low. Most participants (439/565, 77.7%) across the two intervention conditions failed to complete a single module of the program. Some participants (126/565, 22.3%) completed at least part of the program, with only 1.8% (10/565) completing all 14 modules of the program. A Mann-Whitney *U* test indicated no significant difference in the number of modules started between the EFI + *myCompass 2* (mean rank=284.91) and the *myCompass 2* alone (mean rank=281.13) conditions (*U=*39366.50; *z*=−0.32; *P=*.75). There were no differences in the number of modules *completed* between the EFI + *myCompass 2* (mean rank=284.45) and the *myCompass 2* alone (mean rank=281.58) conditions (*U=*39494.0; *z*=−0.29; *P=*.77). Figure 3 presents a cumulative graph comparing the conditions of uptake and adherence to the programs.

### Efficacy (H3 and H4)

There were no significant overall interactions between conditions and measurement occasions for depression (*F*_4,349.97_=1.16; *P=*.33) or anxiety (*F*_4,445.99_=0.12; *P=*.98) based on MMRM analyses. Planned contrasts demonstrated no significant interactions between time and conditions on symptoms of depression or anxiety at posttest or follow-up. As the intervention was not significantly effective overall, we restricted our planned moderation analyses of these outcomes to examine module completion, symptom levels, and basic demographics (age and gender) only. First, we examined those who completed a greater number of modules in the *myCompass 2* program. In MMRM analyses testing the effect of module completion (0 vs 1-14 modules) on mental health outcomes, the three-way interaction between module completion, time, and condition was not significant (PHQ-9: *F*_4,313.66_=0.49; *P=*.74; GAD-7: *F*_4,326.46_=0.31; *P=*.87), indicating no differential effects of the intervention for those who were more engaged. Table 3 provides the mean (SD) for depression and anxiety scores among the completers. We also examined the modification of intervention effects by symptom levels for both GAD-7 and PHQ-9 (none or mild vs moderate symptoms). The effects were only significant for GAD-7, suggesting that people with elevated generalized anxiety disorder symptoms at pretest may have benefitted from the intervention more than those with lower symptoms (GAD-7: *F*_4,332.80_=3.50; *P=*.01). Finally, the three-way interaction between time, condition, and age (PHQ-9: *F*_4,366.09_=1.01; *P=*.40; GAD-7: *F*_4,433.19_=0.99; *P=*.41) and time, condition, and gender (PHQ-9: *F*_4,328.19_=1.63; *P=*.17; GAD-7: *F*_4,348.97_=1.01; *P=*.40) was not significant.

**Table 3:** Observed means and SDs for the secondary outcome measures at pre- and posttest for the trial conditions.

Measure and condition			Measurement occasion												
			Pretest					Posttest					6-month follow-up		
			Participants, n		Value, mean (SD)		Participants, n			Value, mean (SD)		Participants, n			Value, mean (SD)
**Depression (PHQ-9^a^)**															
	EFI^b^ + *myCompass 2*	280		9.82 (2.77)		75			9.00 (5.18)		91			9.34 (5.47)	
	*myCompass 2* alone	285		9.64 (2.70)		84			8.90 (5.12)		108			7.90 (4.68)	
	Attention control	284		9.49 (2.92)		123			8.52 (4.94)		136			8.24 (5.86)	
**Anxiety (GAD-7^c^)**															
	EFI + *myCompass 2*	280		7.63 (3.42)		75			7.17 (4.49)		91			6.98 (4.56)	
	*myCompass 2* alone	285		7.28 (3.27)		84			6.55 (4.51)		108			5.96 (4.53)	
	Attention control	284		7.71 (3.37)		123			6.57 (4.46)		136			6.26 (4.85)	
**General psychological distress (DQ5^d^)**															
	EFI + *myCompass 2*	280		14.50 (2.96)		75			13.89 (4.15)		91			13.98 (4.16)^e^	
	*myCompass 2* alone	285		14.39 (3.07)		81			12.96 (3.74)		105			12.50 (3.95)	
	Attention control	284		14.42 (2.87)		118			13.11 (3.65)		135			12.82 (4.35)	
**Acceptability (UTAUT^f^)**															
	EFI + *myCompass 2*	280		14.49 (3.18)		75			14.16 (3.93)		91			12.84 (3.99)	
	*myCompass 2* alone	285		14.42 (3.01)		81			14.42 (3.25)		105			13.51 (3.69)	
	Attention control	284		14.91 (2.87)		118			14.03 (3.29)		135			13.59 (3.97)	
**Performance expectancy (UTAUT)**															
	EFI + *myCompass 2*	280		13.53 (2.67)		75			13.48 (3.54)		91			12.54 (3.69)	
	*myCompass 2* alone	285		13.41 (2.67)		81			13.86 (3.33)		105			13.14 (3.51)	
	Attention control	284		13.89 (2.70)		118			13.64 (3.10)		135			13.00 (3.66)	
**Effort expectancy (UTAUT)**															
	EFI + *myCompass 2*	280		10.66 (2.19)		75			10.40 (2.28)		91			10.20 (2.47)	
	*myCompass 2* alone	285		10.67 (2.12)		81			10.30 (2.35)		105			10.12 (2.23)	
	Attention control	284		10.54 (1.98)		118			10.49 (2.17)		135			10.37 (2.23)	
**Data security concerns (UTAUT)**															
	EFI + *myCompass 2*	280		7.48 (1.86)		75			6.97 (1.82)		91			6.82 (2.04)	
	*myCompass 2* alone	285		7.30 (1.96)		81			7.26 (1.99)		105			7.25 (1.93)^g^	
	Attention control	284		7.37 (1.85)		118			6.95 (2.07)		135			6.68 (2.16)	
**Days out of role**															
	EFI + *myCompass 2*	280		3.04 (4.49)		75			3.81 (5.68)		91			3.52 (5.85)^h^	
	*myCompass 2* alone	285		4.14 (5.92)		80			3.91 (5.67)		105			3.43 (6.45)	
	Attention control	284		3.62 (5.65)		118			3.66 (6.43)		135			2.97 (5.87)	
**Days cut down**															
	EFI + *myCompass 2*	280		9.01 (8.33)		75			9.19 (8.83)		91			9.12 (9.72)	
	*myCompass 2* alone	285		9.51 (8.65)		80			7.13 (7.32)		105			8.55 (9.05)	
	Attention control	284		7.60 (7.32)		118			7.31 (8.07)		135			8.33 (9.63)	
**Quality of life (EUROHIS-QOL^i^)**															
	EFI + *myCompass 2*	280		24.19 (5.15)		75			24.24 (6.63)^j^		91			24.46 (6.22)^k^	
	*myCompass 2* alone	285		23.57 (5.13)		80			25.15 (5.81)		105			25.40 (6.06)	
	Attention control	284		24.46 (5.68)		118			24.86 (5.97)		135			25.75 (6.26)	
**Suicidal ideation (PSF^l^)**															
	EFI + *myCompass 2*	280		0.96 (1.21)		—^m^			—		91			0.91 (1.21)	
	*myCompass 2* alone	285		0.95 (1.21)		—			—		105			0.76 (1.13)	
	Attention control	284		0.84 (1.09)		—			—		135			0.82 (1.17)	

^a^PHQ-9: Patient Health Questionnaire-9.

^b^EFI: engagement facilitation intervention.

^c^GAD-7: Generalized Anxiety Disorder-7 item.

^d^DQ5: Distress Questionnaire-5.

^e^Pretest to 6-month follow-up versus *myCompass 2* alone (*P*=.03).

^f^UTAUT: Unified Theory of Acceptance and Use of Technology.

^g^Pretest to 6-month follow-up versus attention control (*P=*.01).

^h^Pretest to 6-month follow-up versus *myCompass 2* alone (*P=*.04) and versus attention control (*P=*.03).

^i^EUROHIS-QOL: European Health Interview Survey Quality of Life 8-item index.

^j^Pretest to posttest versus *myCompass 2* alone (*P=*.02).

^k^Pretest to 6-month follow-up versus *myCompass 2* alone (*P=*.02).

^l^PSF: Psychiatric Symptom Frequency scale.

^m^Data for the Psychiatric Symptom Frequency scale measures the items over the previous 6 months; Psychiatric Symptom Frequency scale data were collected at pretest and 6-month follow-up only.

### Secondary Indices of Efficacy (H6)

MMRM analyses showed that there were no significant overall interactions between conditions and over time for any of the factors related to the acceptability of internet-based psychosocial interventions based on acceptance (*F*_4,316.99_=0.39; *P=*.82), performance expectancy (*F*_4,343.41_=0.55; *P=*.70), effort expectancy (*F*_4,357.35_=1.31; *P=*.83), or concerns regarding data security (*F*_4,337.82_=1.68; *P=*.16). Similarly, there were no significant effects on general psychological distress (*F*_4,382.08_=1.31; *P=*.27), disability (*F*_4,361.21_=1.70; *P=*.15), the days out of role (*F*_4,366.45_=1.33; *P=*.26), or overall quality of life (*F*_4,357.55_=1.99; *P=*.10). Suicidal ideation also did not differ between the pretest and 6-month follow-up groups (*F*_2,375.49_=0.70; *P=*.50). Table 3 shows that planned contrasts demonstrated several significant interaction effects between conditions over time, although these were inconsistent across time points and none appeared to be in the expected direction.

### Reasons Given for Nonadherence

A total of 47.1% (128/271) of 271 participants who responded to this question reported that they did not complete their assigned program at posttest. This self-reported rate was similar to those who were automatically recorded to have not started (389/849, 45.8%) or completed (475/849, 55.9%) a single module of their program (Table 2). Table 4 presents the self-reported reasons for not competing with the program for the two intervention groups. Almost half (49/101, 49.5%) of participants from the *myCompass 2* alone (n=49) and EFI + *myCompass 2* (n=52) conditions reported time as an important barrier that prevented them from completing the *myCompass 2* program. Other major barriers included technical issues, simply forgetting to use the program, experiencing difficulties with concentration, or fatigue.

**Table 4:** Coded responses and example quotes for reasons for nonadherence given by participants in engagement facilitation intervention + *myCompass 2* and *myCompass 2* alone conditions^a^.

Themes and subthemes		Example quotes (condition)	Responses coded in theme (n=144), n (%)	Respondents mentioning theme (n=101), n (%)
**Structural barriers**			67 (46.5)	67 (66.3)
	Lack of time or competing demands	“Just time with work and study” (EFI^b^ + *myCompass 2*) “Haven’t found the time” (EFI + *myCompass 2*).	49 (34)	49 (48.5)
	Technical issues	“There were some bugs in some of the questions and answers” (*myCompass 2* alone) “Internet at home stopped working” (*myCompass 2* alone)	18 (12.5)	18 (17.8)
**Physical or mental barriers**			51 (35.4)	51 (50.5)
	Forgot to use program	“Forgot. A more regular prompt would have been beneficial” (*myCompass 2* alone) “Just forgot it was there” (EFI + *myCompass 2*)	21 (14.6)	21 (20.8)
	Fatigue or concentration issues	“I have trouble concentrating for long period of time” (EFI + *myCompass 2*) “No mental energy” (*myCompass 2* alone)	15 (10.4)	15 (14.9)
	Lack of motivation	“It was hard to find the time and the motivation to do so” (*myCompass 2* alone) “I didn’t feel like filling it out especially if I was having a good day” (EFI + *myCompass 2*)	9 (6.3)	9 (8.9)
	Too unwell	“I was right unwell mentally and was more focus (sic) on that than internet based program.” (*myCompass 2* alone)	6 (4.2)	6 (5.9)
**Program barriers**			16 (11.1)	16 (15.8)
	Poor fit of program to needs	“I found the information too general, not really suited to my needs and way too basic. If someone had a serious issue this would not have helped. I found I thought of it as a chore.” (EFI + *myCompass 2*) “It did not offer modules that were useful for my mental health problems” (*myCompass 2* alone)	10 (6.9)	10 (9.9)
	Disliked program	“It seemed repetitive” (EFI + *myCompass 2*). “Bored. Activities were very samey and paint by numbers. Didn’t see the connection between some activities. Needed an overview of what I should be doing and when. Couldn’t see the path I was supposed to be following. Activities didn’t feel specific to me.” (*myCompass 2* alone)	6 (4.2)	6 (5.9)
**Other**			10 (6.9)	10 (9.9)
	Major life events	“Suffered an intense relationship breakdown (10 years) half way through the program” (*myCompass 2* alone)	4 (2.8)	3.9 (4)
	Not accountable	“I found it slipped to the bottom of my to-do list everyday as it did ‘t (sic) have a set time to do it, and no one holding me accountable” (*myCompass 2* alone)	2 (1.4)	1.9 (2)
	Completed it	“Still completing” (*myCompass 2* alone)	2 (1.4)	1.9 (2)
	Miscellaneous (trust and cost)	“Was scared of the cost” (*myCompass 2* alone)	2 (1.4)	1.9 (2)

^a^Responses were coded multiple times into themes (144 codes from 101 responses). Total data (n=101) were from participants from the *myCompass 2* alone (n=49) and EFI + *myCompass 2* (n=52) conditions only. Data from the control group were omitted (n=27).

^b^EFI: engagement facilitation intervention.

## Discussion

### Principal Findings

This study describes the outcomes of the EEPI trial, which involved an RCT of an EFI designed to increase uptake and adherence to a self-guided internet-based mental health program. The EFI was not found to be efficacious in improving the uptake of or adherence to E–MH intervention in this study. These findings are somewhat consistent with those of Lin et al [30], who found that despite their sample having a high acceptance of internet interventions for pain management, the uptake rate of the intervention was only moderate and adherence was very low. In contrast, uptake was high in this study, which likely reflected the minimal effort required to begin the intervention, although adherence was very low. The sample in this study was larger than that of the study by Lin et al [30], which was powered to detect more modest effects of the EFI in the context of a mental health intervention. The EFI used in this study also addressed barriers, in addition to acceptability. However, the results were similar, with no differences in uptake or adherence. The EFI in this study was also unable to significantly improve acceptability of internet interventions. The lack of difference in both uptake and acceptability may be related to ceiling effects, in that most participants were accepting of E–MH interventions and at least clicked through to the intervention. However, the lack of difference in the number of modules started suggests that EFI had minimal effect on both uptake and adherence. The findings do not preclude specific effects of the EFI; for example, some participants who received the EFI may have been motivated to engage more with the intervention, whereas others may have recognized that the intervention was not suitable for them and engaged less. However, this study shows no evidence that this implementation strategy is likely to be effective at the scale of increasing adherence.

In addition, the *myCompass 2* program was not found to be efficacious in improving depression or anxiety in this sample. In contrast, several previous RCTs have demonstrated improvements in depression and anxiety in community-based samples for the original *myCompass* program [37,38]. It is unclear whether the lack of demonstrated efficacy was related to the redesign of the *myCompass 2* program for this study, the characteristics of the sample (moderate depression or anxiety symptoms), the fully web-based nature of the trial (and consequent high attrition), or a combination of these factors. Our planned contrasts indicated that the program may be more beneficial for those with moderate anxiety symptoms. However, this result should be interpreted with caution given that the overall model for efficacy was not significant; we acknowledge that this test was post hoc and could be by chance. Further investigation is warranted to test the conditions under which this program may be effective. There is a possibility that a poor fit between the program and the needs of the participants may have had a negative impact on adherence. Additionally, the broad lack of adherence may have affected our ability to detect efficacy in this study. Nevertheless, module completion did not improve the program’s efficacy, and it was not found to be effective at scale in this real-world community-based trial.

Despite the null effects of the trial, the implications of these findings remain important. There was no evidence that the implementation strategy of educating participants about their need for intervention (feedback on symptoms), benefits, perceived barriers to use, and norms of engaging with psychosocial interventions was effective in increasing engagement (uptake and adherence) with a subsequent E–MH program. Further research might evaluate whether specific components of this EFI may be able to influence the engagement of specific groups of people using factorial experiments or additional qualitative methods. It may also be the case that uptake and adherence are more challenging in the context of potentially complex mental health needs but may be more amenable to intervention in the context of other health problems [28,29].

At the outset of the study, it was clear that adherence to psychosocial interventions is a complex and dynamic behavior [14,20]. People decide not to engage with interventions for diverse reasons, many of which are entirely appropriate [14,20-22]. Targeting or tailoring both implementation strategies and interventions to the needs of an individual may be required to improve engagement. Adaptive interventions tailored to the barriers relevant to the individual, with ongoing check-ins over the duration of the intervention period may be more successful than a one-time, low-intensity strategy. This may be of benefit, as many participants simply noted *forgetting* as a barrier to using the program. Blending human support with a self-guided program may also be beneficial for increasing the uptake of internet interventions [58], although a blended approach would come at the cost of making the intervention less scalable, as human support requires additional resources. Human support for internet-based interventions may be critically important for certain individuals. An overwhelming majority of participants who noted that they did not complete the program believed that a lack of time, or competing demands for their time, was a strong barrier to completing the program. However, having accountability to another person may assist in challenging this barrier [36], as it requires commitment and time to be set aside in advance. Nevertheless, this issue remains complex, as a requirement for human contact may deter some individuals from signing up for such a program.

Importantly, the quality of the therapeutic alliance can influence treatment outcomes [59], regardless of whether it is with a computer or human. Consequently, taking into account human-computer interactions in the design of both the implementation strategy and the psychosocial intervention may also promote engagement by increasing a sense of therapeutic alliance or human connection in the internet-based setting. The co-design of interventions with end users is also imperative to ensure that interventions meet the needs and preferences of those who stand to benefit. Nevertheless, based on the current findings, our partnership with end users was not sufficient to realize the aims of the EFI. The need for psychological services will continue to increase and cannot be fully met by increasing the health professional workforce. Creative and rigorous methods to increase the use of self-guided interventions in the community, for prevention and treatment of health problems, may lead to reduction in disease burden over time.

### Limitations

Although this was one of the first studies to rigorously evaluate an implementation strategy to increase engagement with a self-guided psychosocial intervention, there were some limitations of both the EFI and this study that should be noted.

#### Engagement Facilitation Intervention

First, as noted above, the EFI may have been too brief or insufficiently tailored to the needs of users. Although our development approach involved considerable collaboration with people who had lived experience of depression or anxiety, it is possible that the intensity of the EFI was insufficient or that it did not meet the diverse needs of users in the trial. We also did not assess participants’ engagement with the EFI (eg, how much time they spent on it or if they read or watched the content, or just clicked through the pages). Attrition was the greatest in the EFI condition. It is possible that there was greater disengagement in the study in this condition, which may have occurred if the information from the EFI had a demotivating effect (ie, provided information that the psychological intervention was not of interest to the participant). When educating potential users about psychosocial interventions, there remains a risk of either overwhelming users with information or inadvertently reducing their motivation to engage.

#### Study Design

Overall, the attrition from the study was considerable. Although the study remained well-powered to detect hypothesized effects on uptake and adherence, it was slightly underpowered to examine efficacy outcomes, as the final samples in the active conditions were less than the targets (n=111). Attrition may have also led to biases in the analyses, although rigorous MMRM models were used to account for all available data. Incentives were used to minimize attrition but clearly provided insufficient motivation for most participants. Attrition from fully internet-based trials remains a challenge, which indicates that some form of human contact in a research trial is likely to be necessary to maintain samples over extended periods. Moreover, there were some technical challenges in the delivery of the trial. Participants could not be automatically logged into the *myCompass 2* program and were required to sign up for the intervention as a separate process, which may have led to reductions in uptake for both active conditions and raised the slight possibility of dual accounts that we may not have been able trace (ie, greater adherence than observed). The lack of efficacy of the *myCompass 2* program in this study suggests that the intervention may be better suited to participants with different symptom profiles or that further work is needed to refine the intervention based on the low adherence rates. Nevertheless, the lack of evidence for efficacy did not limit our ability to compare the levels of uptake or adherence within the RCT design of the study.

The use of Facebook or certain imagery in advertisements may have attracted a certain type of user; however, we believe this is not a significant limitation as it reflects a similar process for real-world marketing of internet-based interventions and typical users of internet-based programs, and the promotion of the trial was identical across the three arms of the trial. Participants in the trial may have been aware of their allocation based on the content, despite interventions not being explicitly labeled as active or control. Finally, the composition of the sample was biased toward female participants. Although this imbalance reflects the usage of E–MH programs in the community, it may have limited the generalizability of the results for males.

### Conclusions

Although there is considerable scope for self-guided psychosocial programs to reduce health burdens, the uptake and adherence to these programs in the general population is limited. EFIs have been proposed as a specific strategy to overcome the implementation gap in psychosocial programs. However, this study indicates that the strategy was not effective in the context of an internet-based mental health program based on cognitive behavioral therapy for people with mild-to-moderate symptoms of depression or anxiety. Further research is required to identify implementation strategies that consider the dynamic and complex nature of intervention adherence and minimize the engagement barriers associated with internet-based programs.

## Appendix

Multimedia Appendix 1 CONSORT 2010 (Consolidated Standards of Reporting Trials) checklist for reporting randomized trials.

Multimedia Appendix 2 CONSORT-eHEALTH checklist (V 1.6.1).

## Abbreviations

AFI: acceptance-facilitation intervention
CONSORT: Consolidated Standards of Reporting Trials
DQ5: Distress Questionnaire-5
DSM: Diagnostic and Statistical Manual of Mental Disorders
EEPI: Enhancing Engagement with Psychosocial Interventions
EFI: engagement facilitation intervention
E–MH: e–mental health
EUROHIS-QOL: EUROHIS-Quality of Life 8-item index
GAD-7: Generalized Anxiety Disorder 7-item
MMRM: mixed model repeated measures
NHMRC: National Health and Medical Research Council
PHQ-9: Patient Health Questionnaire-9
RCT: randomized controlled trial
